# A simple new method for aged seed utilization based on melatonin-mediated germination and antioxidant nutrient production

**DOI:** 10.1038/s41598-021-85541-7

**Published:** 2021-03-15

**Authors:** Song Yu, Xuetian Zhu, Helin Yang, Lihe Yu, Yifei Zhang

**Affiliations:** Heilongjiang Higher Educational Key Laboratory for Cold-Regional Crop Cultivation and Germplasm Improvement, Department of Agronomy, Heilongjiang Bayi Agricultural University, No. 2, Xinyang Road, High-Tech Development Zone of Daqing, Daqing, 163319 China

**Keywords:** Plant physiology, Biochemistry

## Abstract

Seed deterioration, coupled with a decrease in nutrients, is unavoidable following long-term storage, and these seeds are therefore used as livestock fodder. Here, we developed a simple, rapid and efficient method of producing high amounts of antioxidants from deteriorated seeds via melatonin-induced germination. Legume seeds were subjected to high humidity at 55 °C for 12–36 h to obtain aged seeds with a 40% germination rate and severely reduced antioxidant nutrition (total phenolics content, ferric reducing power and 1,1-diphenyl-2-picryhydrazyl (DPPH) radical scavenging capacity). Aged seeds were then treated with 0.1 mM melatonin, resulting in the production of sprouts with a higher total phenolics content (fivefold), greater ferric reducing power (sevenfold) and greater DPPH radical scavenging capacity (twofold) compared to the aged seeds. These findings suggest that melatonin treatment efficiently converted aged seed reserve residues into antioxidant nutrients, providing an alternative use for deteriorated seeds in food production.

## Introduction

Free radicals produced by the body are neutralized by antioxidants, thereby alleviating oxidative damage^1^. Proper balance between free radical production and scavenging is therefore necessary for optimum physiological function; therefore, external antioxidants are often used to reduce damage^2^. For example, certain antioxidants such as polyphenol, which plays an important role in regulating metabolism and alleviating chronic disease^3^, cannot be biosynthesized by the body and are only acquired through our diet^2^.

Legumes are an important source of starch, protein and lipids^4–7^, as well as containing high levels of polyphenol^6^ and vitamin C^8^. For example, soybeans [*Glycine max* L., Merr.], which are widely used in the food industry, contain a high amount of bioactive molecules such as polyphenol^9,10^.

Legume seed germination for sprout production was introduced by the Egyptians thousands of years ago^11^, and involves soaking, draining then leaving the seeds until they germinate and begin to sprout^12^. Germination drives some of the seed reserves towards the synthesis of new metabolites, leading to profound changes in the accumulation of nutrients in the developing embryo^13^. Germinated legume seeds in particular produce a large amount of polyphenol in the sprouts^6,12,14^.

However, seed deterioration is a common natural process in agricultural production, and respiration results in the accumulation of damaged DNA, lipids, and protein^15,16^ as well as loss of nutrient reserves^17–19^ during ageing or adverse environmental conditions^20,21^. Seed deterioration in soybean is easily observed both in the field^22^ and at the storage stage^23^. Data also suggest that the reduction in antioxidant enzyme (e.g. catalase) activity^24^ and decrease in non-enzymatic antioxidants (e.g. glutathuione and α-tocopherol) can be monitored in deteriorated seeds due to free radical accumulation^25–27^. Thus, a number of priming methods have been developed for the restoration of aged seeds^28^. For example, melatonin (*N*-acetyl-5-methoxy-tryptamine), a well-known animal hormone, has also been discovered in plants and was found to significantly restore vitality in aged seeds^29–31^. Moreover, melatonin was also found to play a signaling role during seed germination under both stressful^32–35^ and stress-free conditions^36,37^.

In this study, four types of deteriorated bean seeds (yellow and black soybean, mung and red bean) were obtained via artificial ageing treatment. Melatonin was then used to restore vitality and promote sprout production, resulting in a large content of antioxidant nutrients. This method uses cheap and readily-available aged legume seeds, and does not require a lot of time or complicated techniques. It can therefore be widely used to address antioxidant nutrient deficiency around the world.

## Materials and methods

### Seeds

Seeds of yellow soybean (*Glycine max* (Linn.) Merr.), black soybean (*Glycine max* var.), mung bean (*Vigna radiata* (Linn.) Wilczek.) and red bean (*Vigna angularis* (Willd.) Ohwi et Ohashi) were obtained from a seed distributor in Daqing city, China. They were sown in plastic boxes and placed in a thermostat-controlled seed germination incubator (LD-330, Laiende, China) during the entire germination period. Prior to the germination test, accelerated ageing treatment was conducted in a thermostat-controlled seed ageing cabinet (LH-150S, Shanghaiqixin, China). The seeds were placed in each individual “ageing box” compartment and deionized water was added, and then subjected to 55 °C (low aging treatment) or 62 °C (high aging treatment) for 12 h (yellow and black soybean) or 36 h (mung bean and red bean) to obtain artificially aged seeds with a germination rate of approximately 40% (low aged seeds) or 10% (high aged seeds), respectively. After ageing treatment, the seeds were collected and dried at 35 °C for 24 h then moistened with tap water for 6 h prior to all subsequent treatments.

### Seed germination experiments

Seeds were divided into four groups for the germination experiments: a control group (no ageing treatment), aged seeds, and melatonin-treated seeds with and without ageing treatment. Seeds were then soaked in tap water (unaged and aged seeds) or melatonin solution (0.1 mM; unaged and aged seeds) for 8 h, respectively, washed then transferred to plastic boxes containing a layer of fine sand (2 cm depth). Germination tests were carried out in a seed germination incubator (parameters were set as: 25 ± 1 °C and 70% humidity) for five days under dark conditions. Germinated seeds and sprouts were then collected and assayed for total phenolics, ferric reducing power and 1,1-diphenyl-2-picryhydrazyl (DPPH) scavenging capacity as described below. All assays were replicated at least three times to minimize experimental errors, and each replicate involved 100 seeds for each germination test.

### Seed germination assay

When the length of the seed radicle was at least 1 mm, the seeds were classified as having germinated. The number of germinated seeds was counted three times (8 h intervals) per day then the germination rate (GR) was calculated as the percentage of seeds that germinated during the first five days after sowing.

### Total phenolics assay

Samples (0.5 g) of bean seeds and 5-day-old sprouts were collected from unaged and aged seeds for the total phenolics assay. Total phenolics were measured using Folin–Ciocalteu reagent^38^ with some modifications^39^ then absorbance was recorded at 760 nm using a UV–Vis spectrophotometer (Shimadzu) and the results were expressed as gallic acid equivalents.

### Antioxidant capacity assay

For the antioxidant capacity assay, seeds were ground into a powder using a Waring blender (ZG-TJ01, Ningbo, China) prior to solvent extraction, and sprouts were ground to a powder in liquid N_2_ using a mortar and pestle. Approximately 500 mg of seed/sprout powder was then transferred to 0.1 L of 80% (w/v) methanol–water solution, and incubated at room temperature for 4 h in the dark. Extracts were then filtered, the filtrates from each replicate were pooled, and the solvent was discarded under a vacuum at 45 °C using a rotary evaporator. Crude extracts were then stored in a desiccator at 4 °C for subsequent antioxidant capacity analysis using the DPPH radical scavenging method^40^ and ferric reducing ability of plasma (FRAP) assay^41^.

The DPPH radical scavenging capacity assay was carried out using 2.95 mL of 60 μM DPPH and 50 μL of extract sample. Absorbance was then read at 517 nm and the readings were compared with the controls, which involved 50 μL of distilled water instead of extract. The % inhibition of DPPH was then calculated using the followed formula: % inhibition = [(A_control_ − A_sample_)/A_control_] × 100.

The FRAP solution for the ferric reducing power assay was prepared by diluting 10 mM of tripyridyltriazine and 20 mM of FeCl_3_ in 100 mM of pH 3.6 sodium acetate buffer at a ratio of 1:1:10. Standards or extracts (both 100 µL) were then mixed with 2.9 mL FRAP reagent and the absorbance at 593 nm was monitored after samples were allowed to react for 30 min at 37 °C. Data were expressed as mmol FeSO_4_ g^−1^ sample.

### Data analysis

All experiments were conducted in a completely randomized design, with three replicates per treatment. All data were analyzed using Duncan’s multiple range test using SPSS 13.0 software (IBM Cop., Armonk, NY, USA) at *p* ˂ 0.05.

## Results

### Effects of melatonin on aged seed germination

**Figure 1:**
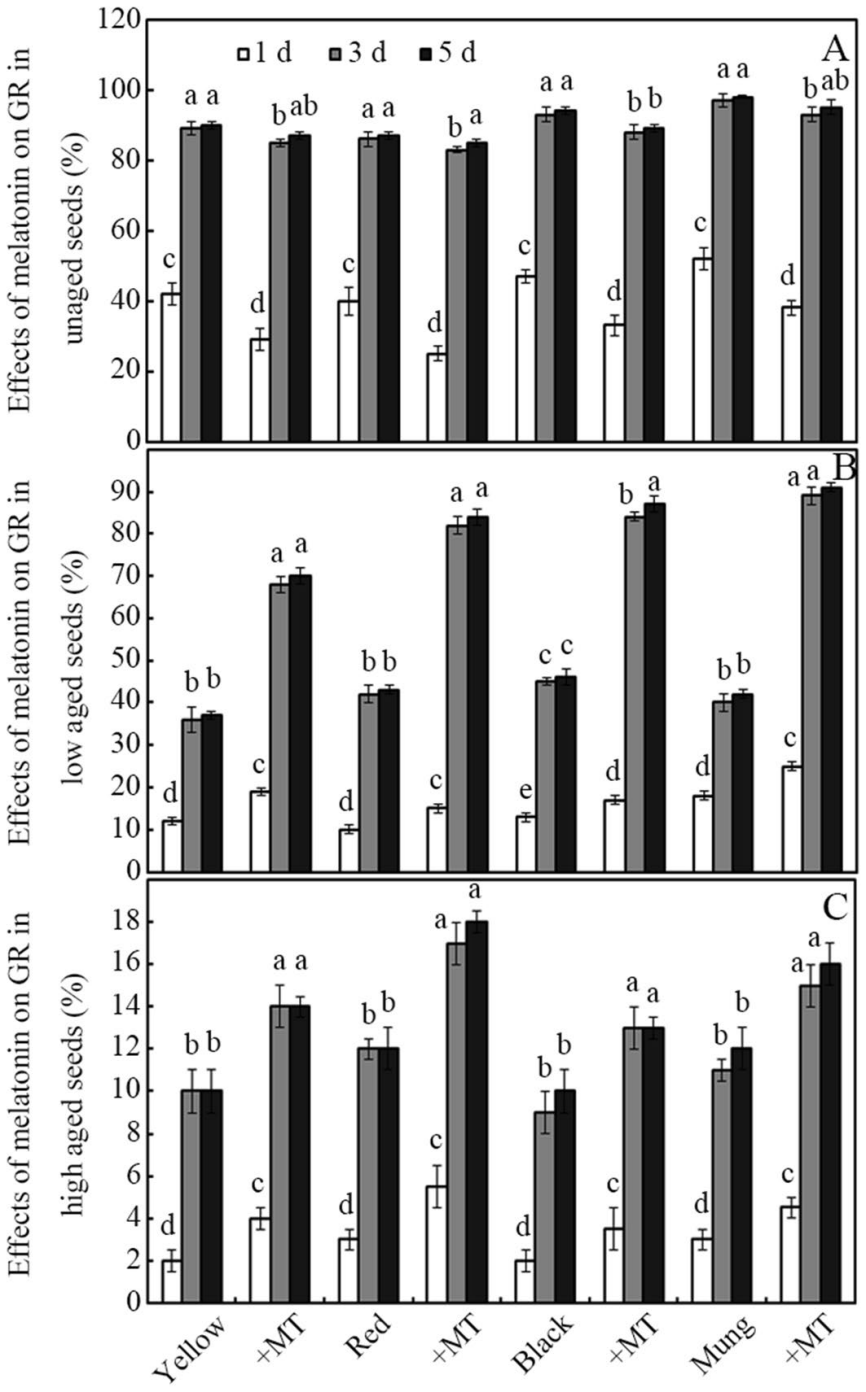
Effects of melatonin on aged seed germination. Recovery effects of melatonin on the germination rate (GR) in unaged (**A**), low aged (**B**) and high aged (**C**) bean seeds imbibed for 5 days. Bars represent standard deviations of the mean (*n* = 3). Means within each bean type followed by the same letter are not significantly different (*p* ˂ 0.05). MT, melatonin; Yellow, yellow soybean; Red, red bean; Black, black soybean; Mung, mung bean.

Treatment with melatonin slightly inhibited or delayed the germination of unaged seeds compared with the untreated control (Fig. 1A). Ageing treatment significantly impaired seed germination (Fig. 1B,C), with 5-day germination rates (GR) of 36–45 and 9–12% in the low and high aged beans, respectively (Fig. 1B,C; *p* ˂ 0.05). However, ageing treatment-impaired germination was profoundly reversed by melatonin, and compared with the aged seeds, treatment with 0.1 mM melatonin significantly increased the GR by approximately 123 and 36% in the low and high aged mung bean seeds, respectively (Fig. 1B,C; *p* ˂ 0.05). Similar changes were also observed in the yellow soybean, red bean and black soybean seeds (Fig. 1B,C; *p* ˂ 0.05).

### Total phenolics and antioxidant capacity in the seeds and sprouts

The total phenolics (TP) content was compared among unaged (control) seeds, aged seeds and sprouts germinated from unaged and aged seeds (Table 1). As shown in Table 1, the aged seeds exhibited a lower TP compared with the unaged seeds, with a decrease of approximately 68, 61, 65 and 59% in the yellow soybean, black soybean, red bean and mung bean, respectively (Table 1; *p* ˂ 0.05). However, melatonin-mediated germination profoundly enhanced TP accumulation in the sprouts (Table 1), with an increase in the yellow soybean sprouts of approximately 2.1- and 11-fold compared with the unaged and aged seeds, respectively (Table 1; *p* ˂ 0.05). Similar changes were also observed in the other bean seeds (Table 1). Overall, the TP contents of both the seeds and sprouts were in the order of yellow soybean < mung bean < red bean < black soybean (Table 1). In addition, germination significantly increased the TP content, and this was further enhanced by melatonin treatment in germinated seeds and sprouts compared with the unaged seeds (Table 1; *p* ˂ 0.05), with significant increases of approximately 20, 12, 14 and 15% in germinated seeds and sprouts from yellow soybean, black soybean, red bean or mung bean, respectively (Table 1; *p* ˂ 0.05).

**Table 1:** Total phenolics contents.

	Yellow soybean	Black soybean	Red bean	Mung bean
Unaged seeds	1.12 ± 0.13d	2.94 ± 0.18c	2.05 ± 0.15c	1.45 ± 0.11c
Unaged sprouts	5.24 ± 0.18b	7.18 ± 0.23b	5.96 ± 0.12b	5.42 ± 0.09b
Unaged sprouts + MT	6.31 ± 0.21a	8.03 ± 0.09a	6.79 ± 0.24a	6.22 ± 0.31a
Aged seeds	0.36 ± 0.04e	1.15 ± 0.09d	0.72 ± 0.13d	0.59 ± 0.04d
Aged sprouts + MT	4.67 ± 0.28c	6.75 ± 0.43b	5.81 ± 0.25b	5.16 ± 0.18b

The total phenolics content (mg gallic acid g^−1^ dry weight) was measured in control and aged bean seeds and sprouts germinated from melatonin-treated aged seeds. Means within each column (n = 3) followed by the same letter are not significantly different (*p* < 0.05). “(un)aged sprout” refers to the germinated seed and sprout from an (un)aged seed.

*MT *melatonin.

Aging treatment also significantly reduced the ferric reducing power of the unaged (control) seeds (Table 2) by approximately 57, 55, 62 and 60% in the aged yellow soybean, black soybean, red bean and mung bean, respectively (Table 2; *p* ˂ 0.05). However, the ferric reducing power was greatly enhanced after sprouting, increasing by approximately 5.5- and 1.9-fold in the black soybean compared to the aged and unaged seeds, respectively (Table 2; *p* ˂ 0.05). Overall, the ferric reducing power of both the seeds and sprouts was also in the order of yellow soybean < mung bean < red bean < black soybean (Table 1). In addition, germination significantly increased the ferric reducing power, and this was further enhanced by melatonin treatment in germinated seeds and sprouts compared with unaged seeds (Table 1; *p* ˂ 0.05), with significant increases of approximately 17, 9, 7 and 12% in germinated seeds and sprouts from yellow soybean, black soybean, red bean or mung bean, respectively (Table 1; *p* ˂ 0.05).

**Table 2:** Ferric reducing power.

	Yellow soybean	Black soybean	Red bean	Mung bean
Unaged seeds	8.2 ± 0.5d	18.8 ± 1.1e	14.4 ± 1.2d	10.8 ± 0.9d
Unaged sprouts	35.5 ± 1.7b	63.2 ± 2.3b	48.9 ± 1.5b	41.7 ± 1.2b
Unaged sprouts + MT	41.5 ± 1.4a	68.8 ± 2.8a	52.3 ± 0.6a	46.8 ± 1.4a
Aged seeds	3.5 ± 0.3e	8.5 ± 0.5d	5.5 ± 0.4e	4.3 ± 0.2e
Aged sprouts + MT	31.7 ± 1.6c	55.1 ± 3.2c	43.7 ± 1.8c	37.3 ± 2.1c

Ferric reducing power (mmol Fe^2+^ g^−1^ dry weight) was measured in control and aged bean seeds and sprouts germinated from melatonin-treated aged seeds. Means with the same column (n = 3) followed by the same letter are not significantly different (*p* < 0.05). “(un)aged sprout” refers the germinated seed and sprouts produced from (un)aged seed.

*MT* melatonin.

Similarly, the sprouts exhibited a greater DPPH radical scavenging capacity (DRSC) compared to the aged and unaged (control) seeds (Table 3). Black soybean extracts exhibited the greatest inhibitory effects compared to the remaining three beans, both the seeds and sprouts (Table 3). However, ageing treatment significantly reduced the DRSC, by approximately 54, 48, 46 and 50% in the aged yellow soybean, black soybean, red bean and mung bean, respectively, compared with the unaged seeds (Table 3; *p* ˂ 0.05). However, this ageing-impaired DRSC was significantly reversed by melatonin-induced seed sprouting (Table 3), with an increase of 246, 140, 155 and 191% in sprouts of yellow soybean, black soybean, red bean and mung bean, respectively, compared to respective aged seeds (Table 3; *p* ˂ 0.05). In addition, germination increased the DRSC, and this was further enhanced by melatonin treatment in germinated seeds and sprouts compared with unaged seeds (Table 1; *p* ˂ 0.05), with increases of approximately 11, 5, 5 and 7% in germinated seeds and sprouts from yellow soybean, black soybean, red bean or mung bean, respectively (Table 1; *p* ˂ 0.05).

**Table 3:** DPPH radical scavenging capacity.

	Yellow soybean	Black soybean	Red bean	Mung bean
Unaged seeds	44.5 ± 2.1d	73.2 ± 1.4c	61.5 ± 2.1c	52.8 ± 1.9d
Unaged sprouts	75.8 ± 1.6b	92.2 ± 0.4b	87.8 ± 1.2b	82.3 ± 2.7b
Unaged sprouts + MT	83.8 ± 1.3a	96.6 ± 0.8a	92.6 ± 1.7a	88.4 ± 1.8a
Aged seeds	20.6 ± 1.1e	38.2 ± 1.6d	33.4 ± 1.8d	26.6 ± 1.4e
Aged sprouts + MT	71.2 ± 2.2c	91.5 ± 2.4b	85.3 ± 1.7b	77.5 ± 1.8c

DPPH radical scavenging capacity (% inhibition) was measured in control and aged bean seeds and sprouts germinated from melatonin-treated aged seeds. Means within the same column (n = 3) followed by the same letter are not significantly different (*p* < 0.05). “(un)aged sprout” refers to the germinated seed and sprout from an (un)aged seed.

*MT* melatonin.

## Discussion

Germination has a profound effect on antioxidant biosynthesis in seeds^12,13^. In this study, the germination rates of the control bean seeds (yellow and black soybeans, red and mung beans) decreased from an average of 90 to 40% (low aged seeds), but even as low as 10% (high aged seeds) after artificial ageing treatment (Fig. 1;* p* ˂ 0.05). Ageing treatment therefore profoundly inhibited seed germination, as in seeds of other crops^42^. Moreover, aging treatment also caused a severe decrease (more than 50%) in the phenolics content and antioxidant capacity (ferric reducing power and DPPH radical scavenging capacity) even in low aged bean seeds (Tables 1, 2, 3), which is partly in line with previous reports showing a rapid decrease in the GSH and vitamin E content in aged crop seeds^26,27^. These findings also suggest that the amount of antioxidant nutrients gained from aged seeds via direct consumption is very low.

As shown in Fig. 1, 0.1 mM melatonin delayed or slightly inhibited the germination of unaged bean seeds, which is in agreement with a previous report showing inhibition of stevia seed germination following treatment with a high concentration (more than 0.1 mM) of melatonin^36^. However, the ageing-induced loss in germination ability was significantly reversed by treatment with melatonin (Fig. 1;* p* ˂ 0.05), a healthy and non-toxic antioxidant^43^. This is in accordance with previous reports whereby melatonin was found to improve germination in aged maize seeds^29,30^. Melatonin, which is an important requirement of the human diet^44^, is found in a number of edible seeds^45,46^, and can also be synthesized in germinated legume seeds and sprouts^47^. Melatonin is therefore a safe reagent for improving germination in aged seeds.

As shown in Tables 1, 2, 3, germination increased the total phenolics accumulation and antioxidant capacity and this was further enhanced by melatonin in sprouts from unaged bean seeds. Interestingly, melatonin treatment also caused increases in the total phenolics content, ferric reducing power and DPPH radical scavenging capacity in sprouts germinated from aged seeds, with increases of at least five-, seven- and two-fold compared to aged seeds, respectively (*p* ˂ 0.05). Germination of aged bean seeds via melatonin restoration therefore has a significant effect on antioxidant nutrient accumulation, which is partly in accordance with a previous report, whereby melatonin improved germination and enhanced the antioxidant capacity of the ascorbate–glutathione system in aged oat seeds^31^. In general, aged seeds are unable to germinate and produce healthy seedlings due to reserve shortages^18^; thus, these seeds are used as animal fodder due to their low nutrient (e.g. protein and starch) content^17–19,26,27,48^. However, as shown here, melatonin-induced sprouting resulted in the conversion of reserve residues into antioxidant nutrients, increasing the overall health benefits of the previously aged seeds^49,50^. In addition, the taste of the resulting bean sprouts is more favorable than that of the aged seeds^11^. Melatonin-induced sprouting of aged seeds therefore results in a functional food product, highlighting the potential applicability of this new method in food production. However, despite our findings, some problems remain. For example, the germination rate of severely aged seeds (e.g. aged seeds with a 10% germination rate) could not be restored to a high level by melatonin, suggesting that this method can only be used with less aged bean seeds.

Numerous data suggest that melatonin promotes seed germination^29–37^. For example, our previous study showed that melatonin priming recovered germination of aged maize seeds by increasing antioxidant enzyme activities^29^. Furthermore, Su et al.^30^ and Yan et al.^31^ addressed the possible mechanisms at a transcriptomic and proteomic levels, respectively, confirming the effect of melatonin. Compared with aged seeds, melatonin can also improve the germination ability of unaged seeds under both stressful^33–35^ and stress-free conditions^36,37^, improving antioxidant enzyme (e.g. SOD and CAT) activities^32,33^, but also enhancing non-enzymatic antioxidant (e.g. total phenolics and ascorbate acid) accumulation^33,35,36^. In this study, compared with aged seeds, melatonin also promoted phenolics nutrient accumulation in unaged bean seeds (Table 1). Antioxidant nutrient accumulation can be partly attributed to melatonin-mediated protein biosynthesis^32–34^ and starch degradation^32^ in the germinated seeds, suggesting that antioxidant nutrient synthesis is required for melatonin-mediated germination in both aged and unaged seeds.

Overall, the results of this study suggest that melatonin can profoundly improve the germination rate and promote sprout production in aged legume seeds. Compared with the aged seeds, melatonin-induced sprouting resulted in rapid conversion of reserve residues into highly valuable bioactive antioxidant nutrients (e.g. phenolics). Since melatonin is an easily acquired natural antioxidant, this method provides an alternative approach for the utilization of deteriorated seed resources.
